# The complete mitochondrial genome of Daurian jackdaw (*Corvus dauuricus*)

**DOI:** 10.1080/23802359.2019.1704191

**Published:** 2020-01-08

**Authors:** Chao Zhao, Huashan Dou, Pengfei Du, Zhao Liu, Lei Zhang, Honghai Zhang

**Affiliations:** a College of Life Science, Qufu Normal University, Qufu, P. R. China;; b Hulunbuir Academy of Inland Lakes in Northern Cold & Arid Areas, Hulunbuir, P. R. China

**Keywords:** *Corvus dauuricus*, mitochondrial genome, phylogenetic tree

## Abstract

In this study, the complete mitochondrial genome of Daurian jackdaw (*Corvus dauuricus*, Pallas, 1776) was sequenced and deposited to GeneBank for the first time using muscle tissue. This mitochondrial genome is a circular molecule of 16921 bp in length and sequence analysis showed it contains 2 rRNA genes, 22 tRNA genes, 13 protein-coding genes and D_loop. The phylogenetic analysis basis of 12 protein-coding genes except for *ND6* gene of 13 species shows that most of the genus of *Corvus* were grouped into two clades, and *C. dauuricus* was basal to all other *Corvus*.

The *Corvus dauuricus* is classified under order Passeriformes, family Corvidae and genus *Corvus*. It is found in the east of Palearctic region, including the areas from Central Siberia to Russian Far East, China and Japan, and it feeds on invertebrates, fruit and seeds (Haring et al. 2007).

In this study, the tissue sample of *C. dauuricus* was catch from Hulun Lake National Nature Reserve, Inner Mongolia, China, and the geo-spatial coordinates are 48°22′19″N latitude and 117°32′20″E longitude. The sample was store in the Animal Specimen Museum of Qufu Normal University, Qufu, Shandong, China with the accession number is QFA20180001. All sampling procedures and experimental manipulations held the proper permits. After manual annotated, the mitochondrial genome was deposited in GeneBank with the accession number MN735458.

The complete mitochondrial genome of *Sorex minutissimus* is a double-circular DNA of 16,921 bp in length and containing 13 protein-coding genes, 22 tRNA genes, 16S rRNA, 12S rRNA and D_loop. Among this gene, *ND6* and 8 tRNA (tRNA*^Asn^*, tRNA*^Ser^*, tRNA*^Ala^*, tRNA*^Glu^*, tRNA*^Cys^*, tRNA*^Tyr^*, tRNA*^Pro^* and tRNA*^Gln^*) genes encoded in L-strand and other genes encoded in H-strand. The percent of base composition is 30.8% for A, 24.9% for T, 14.7% for G, 29.7% for C and the percentage of A and T (55.7%) is higher than G and C (44.3%). This genes arrangement is similar to other *Corvus*, such as *C. coronoides* (Sarker et al. 2017), *C. splendens* (Krzeminska et al. 2016), and *C. hawaiiensis* (Hoeck et al. 2015).

Phylogenetic analysis of 13 Corvidae species (include 10 *Corvus* species), 2 Callaeidae species, and 1 Dicruridae species were analyzed using the maximum likelihood (ML) and the Bayesian inference (BI) methods based on the 12 protein-coding genes except *ND6,* and *Accipiter gentilis* (NC011818) was used as an outgroup. The mode of GTR + I + G was selected as the best-fitting nucleotide substitution mode according to the AIC criterion by MrModeltest 3.7 (Nylander 2004). ML and BI analysis with a bootstrap test of 100 replicates by PAUP 4.0 (Swofford 2002) and MrBayes (Ronquist and Huelsenbeck 2003) was run for 1,000,000 generations used this mode, respectively.

The phylogenetic trees generated from ML and BI methods have the same topologies (Figure 1), and three major phyletic lineages were present in *Corvus*. Most of the *Corvus* were grouped into two clades, and *C. dauuricus* was basal to all other *Corvus*. Furthermore, the phylogenetic relationship of *C. dauuricus* and another *Corvus, C. monedula* were controversial in the past, and the discussion were focused on whether they are two distinct species (Haring et al. 2007). The other Corvidae species were grouped with *Corvus* species and Dicruridae was revealed as a sister group of Corvidae. We expect the data of present study to provide a useful for further research and phylogenetic relationship of *Corvus*.

**Figure 1.**
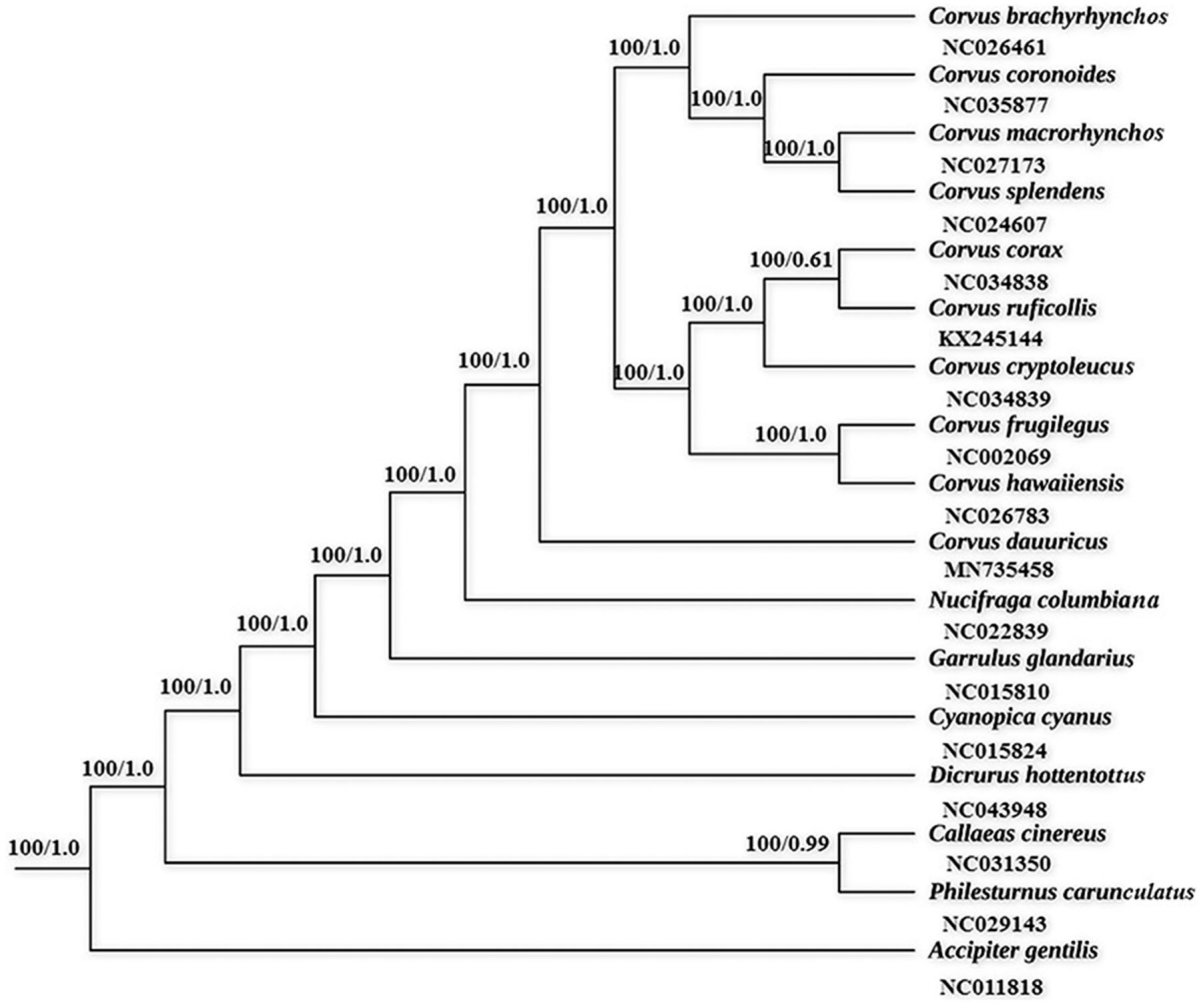
Phylogenetic tree of 16 species was obtain from maximum-likelihood (ML) and Bayesian phylogenetic inference (BI) method based on 12 protein-coding genes, the ML bootstrap proportions and BI posterior probabilities are shown on the nodes. The species accession numbers were downloaded from GenBank are *C. brachyrhynchos* (NC026461), *C. coronoides* (NC035877), *C. macrorhynchos* (NC027173), *C. splendens* (NC024607), *C. corax* (NC034838), *C. ruficollis* (KX245144), *C. cryptoleucus* (NC034839), *C. frugilegus* (NC002069), *C. hawaiiensis* (NC026783), *C. dauuricus* (MN735458), *Nucifraga columbiana* (NC022839), *Garrulus glandarius* (NC015810), *Cyanopica cyanus* (NC015824), *Dicrurus hottentottus* (NC043948), *Callaeas cinereus* (NC031350), *Philesturnus carunculatus* (NC029143) and *Accipiter gentilis*, respectively.
